# Assessing hypo-arousal during reward anticipation with pupillometry in patients with major depressive disorder: replication and correlations with anhedonia

**DOI:** 10.1038/s41598-023-48792-0

**Published:** 2024-01-03

**Authors:** Andy Brendler, Max Schneider, Immanuel G. Elbau, Rui Sun, Taechawidd Nantawisarakul, Dorothee Pöhlchen, Tanja Brückl, BeCOME Working Group, A. K. Brem, E. B. Binder, A. Erhardt, J. Fietz, N. C. Grandi, Y. Kim, S. Ilić-Ćoćić, L. Leuchs, S. Lucae, T. Namendorf, J. Pape, L. Schilbach, I. von Mücke-Heim, J. Ziebula, Michael Czisch, Philipp G. Sämann, Michael D. Lee, Victor I. Spoormaker

**Affiliations:** 1 Department of Translational Research in Psychiatry, Max Planck Institute of Psychiatry, Kraepelinstraße 2-10, 80804 Munich, Germany; 2 Max Planck Institute of Psychiatry, Munich, Germany; 3 Department of Cognitive Sciences, University of California, Irvine, USA; 4 Department of Psychiatry, Weill Cornell Medical College, New York, NY USA; 5 Department of Psychology and Behavioral Sciences, Zhejiang University, Hangzhou, China; 6 University Hospital of Old Age Psychiatry, University of Bern, Bern, Switzerland; 7 Department of General Psychiatry 2, LVR-Klinikum Düsseldorf, Düsseldorf, Germany

**Keywords:** Diagnostic markers, Reward

## Abstract

Major depressive disorder (MDD) is a devastating and heterogenous disorder for which there are no approved biomarkers in clinical practice. We recently identified anticipatory hypo-arousal indexed by pupil responses as a candidate mechanism subserving depression symptomatology. Here, we conducted a replication and extension study of these findings. We analyzed a replication sample of 40 unmedicated patients with a diagnosis of depression and 30 healthy control participants, who performed a reward anticipation task while pupil responses were measured. Using a Bayesian modelling approach taking measurement uncertainty into account, we could show that the negative correlation between pupil dilation and symptom load during reward anticipation is replicable within MDD patients, albeit with a lower effect size. Furthermore, with the combined sample of 136 participants (81 unmedicated depressed and 55 healthy control participants), we further showed that reduced pupil dilation in anticipation of reward is inversely associated with anhedonia items of the Beck Depression Inventory in particular. Moreover, using simultaneous fMRI, particularly the right anterior insula as part of the salience network was negatively correlated with depressive symptom load in general and anhedonia items specifically. The present study supports the utility of pupillometry in assessing noradrenergically mediated hypo-arousal during reward anticipation in MDD, a physiological process that appears to subserve anhedonia.

## Introduction

The locus coeruleus (LC)—the primary source of noradrenergic (NA) signaling in the brain—is involved in a myriad of cognitive and affective processes across species^1^. As an accessible surrogate marker of LC activity, pupillometry is gaining increasing attraction^2^. Since the LC-NA system is directly affected by commonly prescribed antidepressants such as venlafaxine and duloxetine^3,4^, the utility of pupillometry for treatment allocation and early response monitoring within subgroups of patients with major depressive disorder (MDD) appears evident. This is further supported by accumulating empirical studies pointing towards altered pupil dynamics in depressed patients.

Recent work on pupillometry has shown that the pupillary light reflex, which is an index of pupil size in response to light, characterized by an immediate constriction followed by a dilation^5^, is altered in depression. It was observed that patients with a MDD diagnosis had an attenuated constriction velocity^6^ and an overall lower constriction change^7–9^ of the pupil in response to light stimuli relative to control participants. Further work has shown evidence for reduced pupil dilation to positively and negatively valenced face stimuli predicting depressive symptoms following a natural disaster^10^, and that, compared to control participants, either increased or decreased pupil dilation to negatively valenced face stimuli predicted recurrence of depressive episodes^11^. Others reported an overall pupil dilation increase to sad faces in children with a family history of depression^12^. In another study, more depressive symptoms were associated with smaller pupil dilation in response to negative prediction errors in the feedback phase of a reward learning task^13^. In addition, the authors showed that in participants with more depressive symptoms, the prediction error-related pupil dilation findings were inversely related to concentration levels of the neurotransmitter choline in the dorsal anterior cingulate^13^, a core brain region of the salience network^14^, which has been implicated in a range of mental disorders including depression^15^. In this context, other studies have shown that MDD patients compared to healthy controls (HC) had smaller bilateral cerebral blood flow increases in anticipation of congruent and incongruent trials in a Stroop task^16^ and in anticipation of calculations in a mental arithmetic task^17^, indicating deficits in anticipatory and preparatory processes when completing cognitive tasks.

Building on this literature, we started to explore the pupil response during a reward anticipation task^18^. Our work was motivated by findings in macaque monkeys, showing a delineated neurophysiological pathway between the anterior cingulate cortex and LC subserving arousal regulation indexed by pupil dilation^19^. In particular, it was demonstrated that macaque monkeys failed to sustain arousal following a reward-predictive cue until reward delivery when the anterior cingulate cortex, which has direct projections to the LC, was lesioned. This failure in upregulating arousal manifested itself in a dip in the pupillary response compared to non-lesioned animals. In our initial validation work^18^, we were able to translate these findings to healthy human volunteers showing that pupil dilation during reward anticipation is associated with activity in the dorsal anterior cingulate cortex and bilateral anterior insular cortex, that as an ensemble constitute the major hubs of the salience network^14^. Moreover, testing these findings in a subsequent observational study in MDD patients, we observed that the extent of pupil dilation during reward anticipation—a 6-s-long interval between the onset and offset of a reward-predicting cue—was negatively correlated to depressive symptom load. A similar negative correlation with depressive symptom load was observed for regions in the salience network, particularly the right insula^20^. In line with the previously discussed animal work^19^, we interpreted this finding as a failure to maintain and upregulate arousal when preparing for the behavioral response to achieve reward in depression. The question to what extent this process is specifically associated with the subjective phenomenon of anhedonia^21,22^ is still open, since our original study^20^ was underpowered to detect small correlations.

Given the replication crisis in adjacent fields of Neuroscience and Psychology^23–25^, which appears to receive less attention in the field of Psychiatry^26,27^, we aimed at replicating the negative correlation between pupil dilation and the number of depressive symptoms within unmedicated depressive patients. For that purpose, we used the exact same task, readouts and analytic approach as in the original work in an independent consecutive subsample recruited from the same population. A replication would increase our confidence that pupillometry has high clinical utility in assessing hypo-arousal in MDD patients.

Furthermore, we extended our findings by capitalizing on the combined sample to test the specificity of the pupillometric results regarding anhedonia-related symptoms compared to other depressive symptoms and how this relates to activity in multiple brain regions of interest. These included the dorsal anterior cingulate and bilateral insula (as core regions of the salience network^14^), the bilateral ventral striatum (i.e., nucleus accumbens), the medial prefrontal cortex and posterior cingulate (as core regions of the task-negative default mode network^28,29^). Moreover, we further examined the specificity of our results for reward anticipation, by additionally analyzing pupillometric measures from other task phases such as fixation and reward consumption.

## Results

### Replication analysis

#### Pupil dilation and depressive symptom load

**Figure 1:**
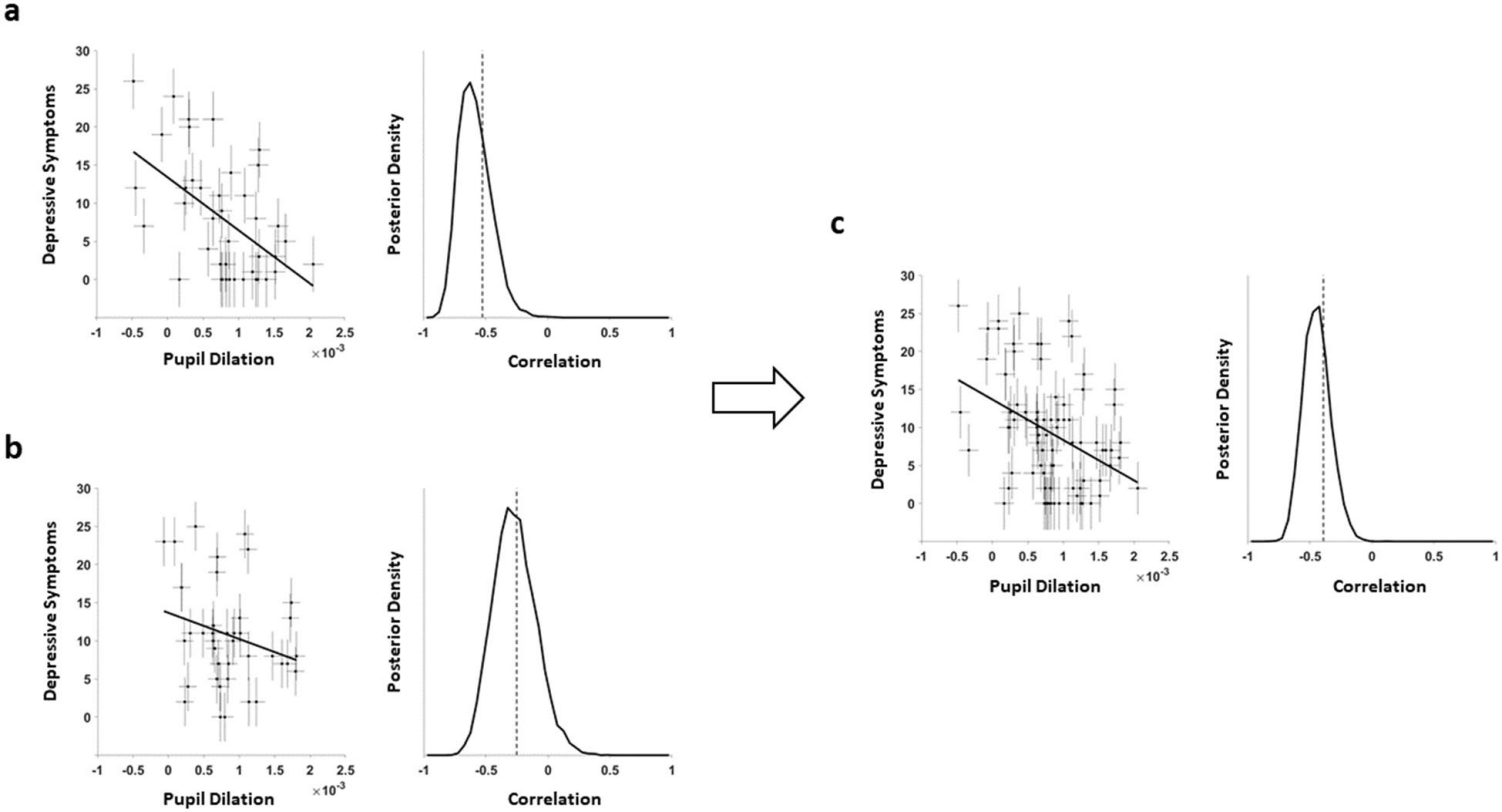
Correlation pupil dilation and number of symptoms for depressed patients. (**a**) original study; (**b**) replication study; and (**c**) combined samples. Estimation of the correlation between pupil dilation during reward anticipation and the number of depressive symptoms taking measurement uncertainty of each measurement into account (represented by vertical and horizontal error bars) including regression lines (**a**, **b**, and **c**, left panels). The Bayesian model estimated the true correlation while accounting for measurement uncertainty by sampling from a multivariate Gaussian distribution, leading to a posterior distribution that indicates the likelihood of the modelled correlation (**a**, **b**, and **c,** right panels).

In the original study, the Pearson correlation was -0.53 (BF_(-)_ = 80.9, Fig. 1a). As our results show, we could replicate this correlation in our new replication sample albeit with a lower effect size (r = -0.26, BF_(-)_ = 14.7, Fig. 1b). Adding the data from both samples yielded a posterior distribution that showed extreme evidence for a negative correlation (r = -0.44, r < 0, BF_(-)_ = 2999, Fig. 1c).

We controlled the correlation of this replication for potential confounding factors in three different ways. First, we computed a partial correlation, controlling for age and gender, resulting in an identical value (r = -0.26). Second, we used a lower estimate (0.70 instead of 0.87) for pupillometry’s test–retest reliability, which resulted in a slightly higher correlation between pupil dilation and symptom load (r = -0.31, BF_(-)_ = 15.4, Figure S1). Finally, to control for the motor response, we used the difference between the reward and verbal feedback stimulus (instead of using the difference between the reward and control stimulus with the latter not requiring a response). This resulted in a lower correlation, but with moderate evidence for it being smaller than zero (r = -0.21, BF_(-)_ = 7.4, Figure S2).

### Extended analyses

#### Pupil Dilation and depressive symptom load across the full sample

**Figure 2:**
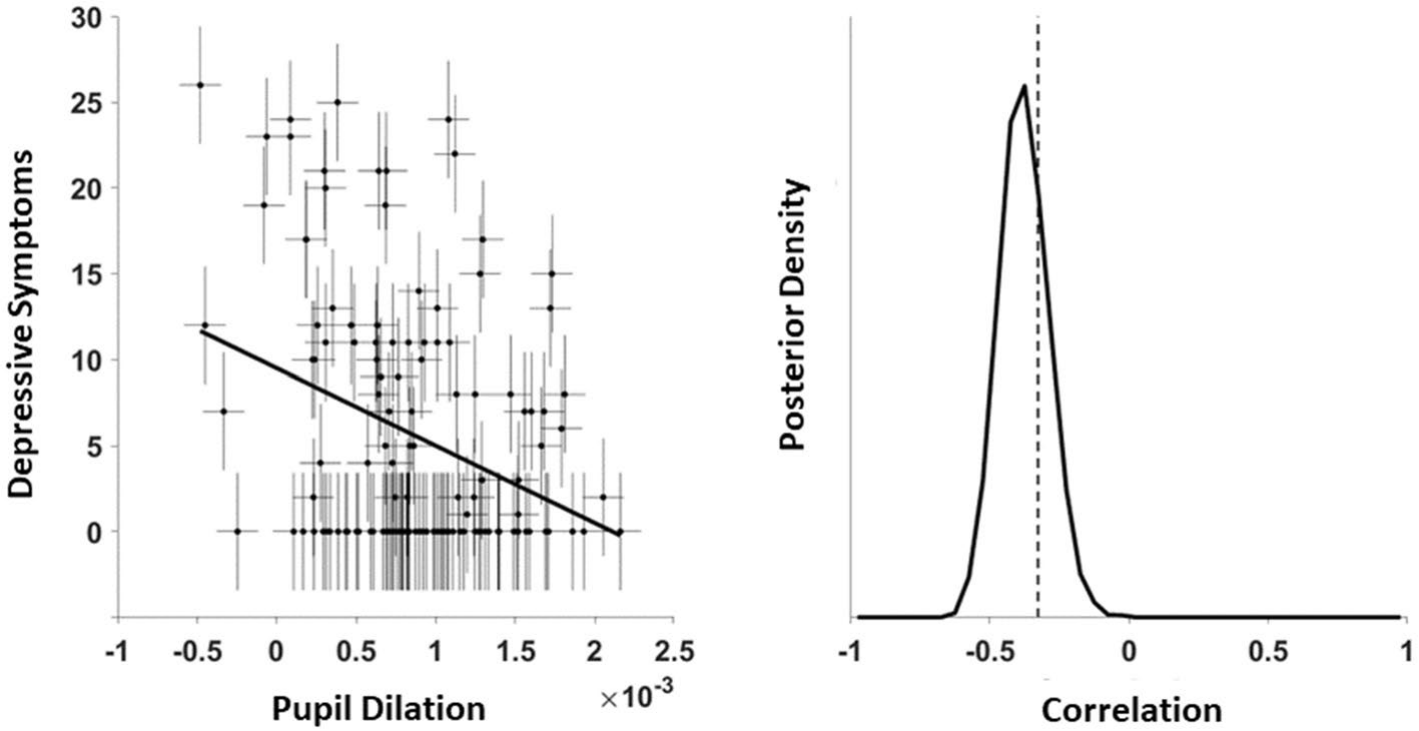
Correlation pupil dilation and number of depressive symptoms for HC and depressed patients. Estimation of the correlation between pupil dilation during reward anticipation and the number of depressive symptoms taking measurement uncertainty of each measurement into account (represented by vertical and horizontal error bars) including regression lines (left panel). The Bayesian model estimated the true correlation while accounting for measurement uncertainty by sampling from a multivariate Gaussian distribution, leading to a posterior distribution that indicates the likelihood of the modelled correlation (right panel).

In order to examine whether the negative correlation between pupil dilation and symptom load could also be observed when analyzing the entire sample including healthy participants, we computed the correlation in all participants from both samples. This resulted in a correlation of -0.38 with extreme evidence in favor of the alternative hypothesis. The model estimation yielded a posterior distribution that was fully below 0, with a BF_(-)_ for r < 0 that was > 1000 (Fig. 2).

### Pupil dilation indexing specific anhedonia symptomatology

To further explore the specificity of our results, we investigated which specific items of the Beck Depression Inventory (BDI-II) correlated with pupil dilation during reward anticipation. Remarkably, we exclusively observed evidence for anhedonia-related items, including ‘loss of pleasure’ and ‘loss of sexual interest’ (see Table 1). Only ‘loss of interest’ as one of the three theoretically proposed anhedonia items^30^ did not correlate with pupil dilation, but instead we observed a correlation with the item ‘loss of energy’.

**Table 1:** Correlation between pupil dilation and BDI-II items.

BDI-II Item	r-value	BF_10_
Sadness	-0.19	1.1
Pessimism	-0.16	0.5
Feelings of failure	-0.18	0.8
**Loss of pleasure**	**-0.26**	**5.9**
Feelings of guilt	-0.03	0.1
Feelings of punishment	-0.04	0.1
Self-dislike	-0.16	0.6
Self-criticism	-0.15	0.4
Suicidal thoughts	-0.15	0.4
Crying	-0.12	0.3
Agitation	-0.09	0.2
Loss of interest	-0.15	0.4
Indecisiveness	-0.17	0.6
Worthlessness	-0.08	0.2
**Loss of energy**	**-0.27**	**9.0**
Changes in sleeping pattern	-0.11	0.2
Irritability	-0.21	1.6
Changes in appetite	-0.02	0.1
Concentration difficulty	-0.20	1.3
Fatigue	-0.19	1.0
**Loss of sexual interest**	**-0.25**	**4.9**
**BDI Anhedonia Score (BDI04, BDI12, BDI21)**	**-0.26**	**5.9**

Correlation between pupil dilation and all 21 individual BDI-II items including the commonly computed anhedonia score consisting of the three BDI-II items: loss of pleasure, loss of interest and loss of sexual interest^30^. Items that show moderate evidence for a correlation, i.e., BF_10_ > 3 highlighted in bold.

### Specificity for pupil dilation during reward anticipation

We further studied the specificity of our results in terms of pupil dilation during reward anticipation in several ways. To show that our pupil results are predominantly reward-related, we correlated the pupil dilations in response to the three stimuli (in contrast to the differential pupil dilation to the reward stimulus minus the control stimulus) with the number of symptoms and the anhedonia subscale score directly. We only observed evidence for correlations of the reward stimulus with number of symptoms (r = -0.28, BF_10_ = 23.4) and anhedonia score (r = -0.25, BF_10_ = 5.7). In contrast, we did not observe any correlations for the neutral stimulus with number of symptoms (r = -0.05, BF_10_ = 0.1) or the anhedonia score (r = 0, BF_10_ = 0.1), neither for the control stimulus with number of symptoms (r = 0.18, BF_10_ = 0.9) or the anhedonia score (r = 0.09, BF_10_ = 0.2).

In order to demonstrate that our pupil measure and its underlying process are specific to reward anticipation and not to other phases of our task, we examined the correlations between depressive symptom load and pupil dilation during the fixation and reward consumption phases. We did not observe any correlations with depressive symptom load when addressing pupil dilation during the fixation phase before presenting the reward stimulus (r = 0.01, BF_10_ = 0.1), the neutral stimulus (r = -0.06, BF_10_ = 0.1), or control stimulus (r = -0.10, BF_10_ = 0.2). Furthermore, no correlation was observed for all these fixation phases combined (r = -0.08, BF_10_ = 0.2). Similarly, the differential pupil score (fixation phase before the reward stimulus minus control stimulus) was not correlated to depressive symptom load either (r = 0.09, BF_10_ = 0.2).

Next, we aimed to find out whether pupil dilation during reward consumption showed a similar response as we observed during reward anticipation, for which we analyzed all trials in which there was a reward consumption, that is, when participants received a reward (i.e., all trials with quick enough responses in the main monetary reward condition receiving a green euro symbol and in the neutral feedback condition receiving a green checkmark symbol). Here again, we did not observe any correlation between pupil dilation and depressive symptom load for the monetary reward condition (r = 0.11, BF_10_ = 0.3), nor for the neutral condition (r = 0.09, BF_10_ = 0.2).

Finally, we show the time courses for pupil size and dilation of the 6 s anticipation window of the reward stimulus (i.e., mean over all 10 trials) for healthy controls and depressed participants in comparison. These are shown for all depressed patients including those who have no acute symptomatology (Figure S3a) and for acutely depressed participants, who reported five or more symptoms in the last two weeks (Figure S3b). In the latter plot a trend for differential pupil responses between groups is better visible, but since only a subset of patients appears to have reward-anticipatory hypo-arousal (e.g., a differential pupil dilation z-score close to or below zero), whole group analyses lack the sensitivity of correlational approaches. This is also reflected in the group comparison results, with anecdotal evidence for overall mean pupil dilation during the 6 s anticipation window being larger for HC than for acutely depressed participants (BF_10_ = 1.46), and no evidence for group differences for HC compared to all depressed participants (BF_10_ = 0.61).

### fMRI results

The region-of-interest (ROI) analyses revealed that pupil dilation during reward anticipation was positively correlated to activity in the right insula (r = 0.26, BF_10_ = 6.6) and negatively correlated to activity in the medial prefrontal cortex (r = -0.23, BF_10_ = 3.1) and the posterior cingulate (r = -0.25, BF_10_ = 4.6). There was no convincing evidence in favor or against a correlation with the dorsal anterior cingulate (r = 0.16, BF_10_ = 0.5) and the left insula (r = 0.18, BF_10_ = 0.8). We found moderate evidence against a correlation between pupil dilation and activity in the left nucleus accumbens (r = 0, BF_10_ = 0.1) and right nucleus accumbens (r = 0, BF_10_ = 0.1). Moreover, the ROI analyses indicated that activity in the right insula in particular was negatively correlated with depressive symptom load (r = -0.28, BF_10_ = 16.8) and the anhedonia score (r = -0.32, BF_10_ = 35.6). There was anecdotal evidence for a negative correlation of activity in the dorsal anterior cingulate with depressive symptom load (r = -0.20, BF_10_ = 1.6) and the anhedonia score (r = -0.21, BF_10_ = 1.4). However, for all other ROIs, there was no evidence for correlations with depressive symptom load or the anhedonia score. There was moderate evidence for group differences between HC and acutely depressed participants in the right insula (BF_10_ = 6.14) and only anecdotal evidence for differences in the dorsal anterior cingulate (BF_10_ = 1.21), with overall reduced activity in these regions during reward anticipation in the acutely depressed participants. We observed no evidence in favor of group differences (instead, this rather represented some evidence against group differences) in the posterior cingulate (BF_10_ = 0.23), medial prefrontal cortex (BF_10_ = 0.40), left nucleus accumbens (BF_10_ = 0.31) and right nucleus accumbens (BF_10_ = 0.21).

## Discussion

The goals of the present study were twofold. First, we aimed to replicate the negative correlation between pupil dilation and symptom load in depressed patients during reward anticipation. To this end, we used the same reward anticipation task and analytic approach as in the original study in an independent sample from the same population. Second, by combining the original and replication samples, we explored the specificity of our pupil measure by examining its relationship with BDI-II items and how this is reflected in brain activity of the salience network. Furthermore, we analyzed the other task phases to probe the robustness of our pupil measure being primarily linked to reward anticipation. In line with our expectations, our results successfully replicate the correlation of interest. Although the effect size of our finding was reduced, modelling the posterior distribution indicated again strong evidence for a negative correlation between pupil dilation and the number of depressive symptoms during reward anticipation. It should be acknowledged that the reduced effect size may to some extent be explained by a phenomenon referred to as the winner’s curse^31^, a term for the more frequently reported overestimation of effect sizes in the discovery sample. Moreover, the negative correlation between pupil dilation and number of depressive symptoms was also observed when healthy controls were added to our analyses. In terms of specificity, our results show that pupil dilation during reward anticipation correlates specifically with anhedonia and lack of energy items of the BDI-II. Furthermore, we did not find any correlations between depressive symptom load and pupil dilation during other phases of our task such as fixation or reward consumption. Overall, the findings of this replication and extension study provide further support for disrupted (reward) anticipatory arousal in depression and highlight the potential of pupillometry to provide a non-invasive biologically-driven measure for heterogeneous samples of MDD patients, with a particular sensitivity to assess anhedonia-related symptomatology.

*To what extent is pupillometry during reward anticipation tracking the LC-NA-system?* Experimental work has shown that pupil dilation constitutes an indirect index of arousal levels produced by widespread NA projections throughout the brain originating from the LC, dependent on state and process^1,32–34^. However, the question remains to which extent we are specifically probing the LC-NA system with pupillometry. In addition to LC, two other brainstem nuclei, namely the pretectal olivary nucleus (PON) and the superior colliculus (SC), have been proposed to be associated with pupillary responses in humans^35^. Although our data does not allow to make any causal claims on the question of cholinergic or noradrenergic mechanisms, pupillometry has shown responsiveness to currently prescribed pharmacological agents depending on their affinity for the LC-NA system. For instance, reboxetine, a selective noradrenalin reuptake inhibitor, and venlafaxine, a serotonin and noradrenalin reuptake inhibitor, affect pupillary properties such as the resting pupil diameter at baseline^3,4,36,37^. In contrast, the selective serotonin reuptake inhibitor paroxetine does not^3^. Moreover, the reward anticipation task is specifically designed to upregulate LC-mediated arousal—also referred to as the alerting system^2^. Taken together, these observations pave the way for embedding pupillometry in clinical schemes, as pupillometry obtained during reward anticipation could support treatment decisions about prescribing a noradrenergically active drug to patients with prominent hypo-arousal on the pupillometry test. Moreover, it could provide a tool to assess the early treatment response by tracking alterations in pupillometric responsiveness.

*How robust is our model to more conservative parameters for uncertainty?* One could argue that our model achieves ‘good’ results, because of our parameter selection. However, several factors point towards the robustness of our model and hence findings. First, we used priors that were all flat. In principle, we could have used the posterior distribution of our original study as prior. However, we refrained from this procedure as this would counteract the idea of an independent replication to a certain extent. Second, even though the test–retest reliability of pupillometry might be considered rather high (0.87), we justified this choice given that this estimate was computed from the data of our sample (see Figure S4). Nonetheless, to completely ensure that our results cannot be explained by this parameter selection, we re-run the model using a lower estimate of the test–retest reliability (0.70). Subsequently, we observed similar results pointing towards our results being robust against variations in measurement uncertainty estimates. In sum, given our choice of using a flat prior and probing the model with different uncertainty estimates, we are confident that changing model parameters leads to less conservative estimates of the effect.

*What is the relevance of the motor response?* This question is more of neuroscientific than of clinical interest—we aimed to adopt a task^38^ for the eye tracker that robustly triggers the LC-NA system and therefore tracks hypo-arousal during reward anticipation. One could argue that our observed effect of the replicated negative correlation between pupil dilation and symptom load is primarily due to the motor response given that we compared the pupillary response to the reward stimulus with the control stimulus (that did not require a motor response). Although this is a fair point, it bears little relevance for a clinical test. If one reliably probes the LC-NA system with our task, then the question of the exact timing in this process, i.e., initial upregulation of arousal, later motor preparation shortly before the response, or a mixture of both, becomes less relevant. Nonetheless, in our study, we could analyze and hence solve this issue by including a third neutral stimulus in our analysis that indicated verbal feedback (i.e., non-monetary reward) upon sufficiently quick motor responses. When re-running the analysis for the difference in pupil dilation between the reward and neutral stimulus, we observed a slightly lower correlation with moderate evidence for it being smaller than zero. This is relevant as this second type of control stimulus could be considered overconservative as it contains verbally ‘rewarding’ feedback. However, even when controlling for motor responses and (verbal) feedback our effect of the replicated negative correlation between pupil dilation and symptom load remains present. This indicates that this effect is largely driven by stimulus type.

*What does our pupillometry test measure?* By further exploring the specificity of our results, we could show that depressive symptom load correlated specifically to pupil dilation during reward anticipation (but not during reward consumption, or fixation). Furthermore, we were able to demonstrate that the pupillometry test appears to be specific for anhedonia items of the BDI-II. Although we did not observe evidence for a correlation with the theoretically proposed anhedonia-item ‘loss of interest’, our results indicate instead moderate evidence for a correlation with the item ‘loss of energy’. Either the ‘loss of interest’ item is phrased too generically or our physiological readout is targeting a certain type of anhedonia that relates especially to lack of energy, potentially capturing a novel subtype of depressed participants. This is further corroborated by our fMRI results: the right insula was positively correlated to pupil dilation and negatively correlated to symptom load and anhedonia. Weaker correlations in the anticipated direction were observed for the dorsal anterior cingulate, but without convincing evidence in favor or against the alternative hypothesis. This is probably a consequence of the chosen peak in the dorsal anterior cingulate (from the stimulus contrast [reward > control stimulus]), which was not optimized for pupil dilation. In our previous work, we have modelled pupil dilation per trial as a parametric modulation to the various stimulus types, and these statistical maps showed different peaks in the dorsal anterior cingulate/premotor area^18^, whereas the peaks in the bilateral insula were more similar. Therefore, it might be a methodological reason why we only observed moderate evidence for correlations in the right anterior insula, although this observation is in line with a few meta-analyses^39–41^. Further studies are needed to disentangle the role of the anterior insula and the salience network in general, its effects on other brain networks and functions^15,41–43^ and how this specifically relates to pupil dilation and hypo-arousal during reward anticipation in MDD. Does the anterior insula reflect stimulus ‘saliency’ in a similar way to pupil dilation or is it a correlate of more integrative, affective processes? This could provide further mechanistical insight into the onset and manifestation of anhedonia on a neural level, thereby providing new directions for its prevention and treatment^44,45^.

To conclude, our study has increased our confidence that pupillometry-based assessed anticipatory hypo-arousal correlates with depressive symptom load in patients with MDD. Furthermore, it provided evidence that certain depressed patients can be characterized by anticipatory hypo-arousal assessed by decreased pupil dilation during reward anticipation, with specificity for anhedonia and lack of energy related items. Therefore, pupillometry’s utility as a clinical test appears to lie in identifying a specific subgroup of patients with anticipatory hypo-arousal subserving anhedonia symptomatology. This may provide valuable information for treatment allocation and examining the early treatment response.

## Materials and methods

### Participants and clinical assessment

A total of 201 new participants were recruited from the Biological Classification of Mental Disorders (BeCOME) study^46^ since the data freeze for our original study^20^ at the Max Planck Institute of Psychiatry in Munich, Germany. After being fully informed about the procedure of the experiment, participants gave their written and informed consent and received reimbursement for participation. All participants were screened on present and past psychiatric and neurological disorders based on anatomical MRI sequences and a general medical interview. Furthermore, an adapted version of the computer-based Munich-Composite International Diagnostic Interview (DIAX/M-CIDI)^47^ was conducted, including additional questions about symptoms of depression and anxiety in the last two weeks. Depression was diagnosed according to the criteria of the Diagnostic and Statistical Manual of Mental Disorders (DSM-IV-TR)^48^. Furthermore, we administered the Beck Depression Inventory (BDI-II)^49^ and evaluated in addition to its 21 items, the commonly reported anhedonia score computed based on the summation of BDI-II items 4, 12 and 21^30^.

Six participants on antidepressant medication and 11 participants with missing diagnostic interview data were excluded from the analysis. Furthermore, we excluded 15 participants due to missing pupillometry data and 32 participants, who exceeded our threshold of no more than 15% missing pupil data, as in our original study (see section Pupillometry recording and pre-processing). In total, 52 participants qualified for a diagnosis of full depression of which 12 had not experienced symptoms within the last 12 months leading to the exclusion from our sample as in our original study. Furthermore, we ensured that control participants did not report any symptoms nor qualified for a mental disorder. Subsequently, the final replication sample for analysis consisted of 40 unmedicated depressed participants (age range: 18–61 years, age *M* = 35.3, *SD* = 12.2, 28 women) and 30 healthy control participants (age range: 19–62 years, age *M* = 35.9, *SD* = 13.3, 20 women). For all new additional analyses, we used the full sample of 136 participants, i.e., original and replication samples combined, with a total of 81 unmedicated depressed participants (age range: 18–63 years, age *M* = 35.2, *SD* = 12.8, 55 women) and 55 healthy control participants (age range: 19–62 years, age *M* = 34.1, *SD* = 12.1, 32 women). The study protocol was in accordance with the Declaration of Helsinki and was approved by a local ethics committee (Ludwig Maximilian University of Munich).

### Reward task

**Figure 3:**
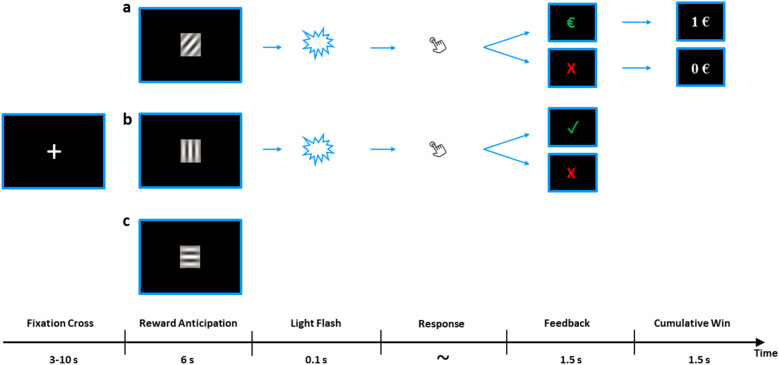
Schematic depiction of the paradigm. Each trial started with a fixation cross, which was followed by one of three Gabor patches corresponding each to a specific condition. Both (**a**) reward condition and (**b**) neutral condition were followed by a light flash to which participants had to respond as fast as possible. Based on the speed of the response a specific feedback was provided indicating if the participant was fast enough or too slow. Given the chance to win 1 euro per trial in the reward condition, the cumulative total win was presented only in this condition. (**c**) was the control condition in which only the Gabor patch was presented and no response was required.

The same adopted reward task^38^ as in the original study^20^ was used. The task was programmed using the Presentation Software (Neurobehavioral Systems Inc., Albany, CA, USA). The stimulus material consisted of the following pictorial stimuli: three isoluminant gabor patches reflecting each one condition, one light flash stimulus, a red cross symbol, a green euro and green checkmark symbol. The exact time course of the task is shown in Fig. 3 below.

Depending on individual reaction times together with an adaptive algorithm that allowed for an approximately 50% success rate per participant across all trials, either positive or negative feedback was displayed for 1.5 s. In the neutral condition, this feedback was either a green checkmark (i.e., fast response) or a red cross (i.e., too slow response). In the reward condition the feedback consisted of a green euro sign if responses were fast enough and a red cross if they were not. It was only in the reward condition where another feedback was shown for 1.5 s that provided the current cumulative total win at that time point in the task. Each participant completed 30 trials in total (i.e., 10 trials per condition) in a pseudo-randomized order.

### Procedure

The task was part of a simultaneous fMRI/pupillometry measurement protocol and thus took place in a 3 Tesla MRI Scanner (MR750, GE, Milwaukee, WI, USA). Prior to being placed in the scanner, participants were seated in front of a computer screen and familiarized with each stimulus type including the study procedure to ensure that they fully understood the task.

### Pupillometry recording and pre-processing

We recorded pupil diameter of the right eye with a sampling rate of 250 Hz using an MRI-compatible eye tracker that was located at the end of the scanner bore (EyeLink 1000 Plus; SR Research, Ottawa, Canada). Eye position was calibrated by means of a nine-point calibration procedure. Pupil data was pre-processed and analyzed using in-house Matlab scripts (MathWorks, Natick, USA), which included the same steps as in our previous study (to rule out the possibility that a changing procedure could explain the results of this replication): First, missing data values due to eye blinks were linearly interpolated from 100 ms before the start of the blink until 100 ms after the end of the blink, which were automatically detected by Eyelink. In order to deal with non-biological outliers, we smoothed the pupil data by using a 200 ms sliding window (100 ms before and after a given data-point), replacing the actual value with the mean value of that window. In a final step, we z**-**transformed the pupil data to account for average variability in pupil size across participants.

Pupil data was discarded as in the original sample based on the following criteria: single trials were discarded if more than 50% of data values were missing, whereas complete data sets were removed if missing data values for the entire session exceeded 15% (*N* = 32). Furthermore, to ensure that pupil data that was analyzed mainly originated from participants looking at the screen, a rectangular gaze window was defined. We computed the median for gaze in x and y directions of the entire 6 s anticipation window yielding two center coordinates per participants. We used those coordinates to determine the average standard deviations for the x–y gaze shift coordinate pair across all participants. Given these parameters, we defined a rectangular gaze window based on 3.3 SD from each participant’s center coordinates. Trials in which participant’s gaze remained outside this window for more than one second were removed.

### Pupillometry analysis

For the replication analysis in this study, as in our original study^20^, we conducted the following analysis steps. The first derivative of the pupil time series data of the reward anticipation phase was computed to obtain pupil dilations for all three conditions (reward, neutral and control condition). To further elucidate the association between depressive symptom load and reward anticipatory arousal, we computed the correlation between the differential mean score of pupil dilation (reward minus control condition) and the M-CIDI item reflecting the number of depressive symptoms within the last two weeks. Correlational analysis between differential mean pupil dilation and the number of symptoms was first conducted only within the depressed participants for the replication and subsequently extended by adding healthy controls. Mean pupil dilations time courses for the 6 s reward anticipation window were compared between healthy controls and depressed participants, as well as between healthy controls and acutely depressed participants (defined as having five or more symptoms within the last two weeks). For further extension analyses, we extracted pupil dilation values for the fixation and reward consumption phases. Here, reward consumption was defined as the feedback phases (with sufficiently quick responses and thus including either a green monetary symbol or green checkmark symbol) in the two reward conditions, that is, (monetary) reward and neutral (verbal feedback) conditions (see Fig. 3).

### Imaging data acquisition and fMRI pre-processing

Functional magnetic resonance imaging (fMRI) data was acquired from a 3 Tesla MRI Scanner (MR750, GE, Milwaukee, WI, USA). We used a 32-channel head coil covering 40 slices (AC-PC orientation of the slices, 3 mm slice thickness, 0.5 mm slice gap, resulting voxel size 2.5 × 2.5 × 3.5 mm^3^, 96 × 96 matrix, in-plane field of view 24 × 24 cm^2^, echo planar imaging (EPI), TR 2.5 s, TE 30 ms, acceleration factor 2). A total of 182 volumes were acquired. Pre-processing of the fMRI data was performed as in our original study^20^ using the Statistical Parametric Mapping software (SPM12, Wellcome Centre for Human Neuroimaging, London, UK, https://www.fil.ion.ucl.ac.uk/spm/). We performed the following pre-processing steps: slice time correction, motion correction through rigid body realignment to the mean volume, spatial normalization using the MNI template. Furthermore, we resampled the data to a voxel resolution of 2 × 2 × 2 mm^3^. Spatial smoothing was performed by applying a Gaussian Kernel with a full width at half maximum: 6 × 6 × 6 mm^3^. The final general linear model included a regressor-of-interest for each of the three stimulus types and several nuisance regressors: six regressors for motion with an additional six regressors for their (absolute) derivatives, three regressors for the signal in the cerebro-spinal fluid and three for the signal in the white matter extracted from their compartment maps using principal component analysis. As in our previous work, we extracted the data from regions-of-interest of the salience network, the default mode network and the ventral striatum: the dorsal anterior cingulate, the left and right anterior insula, the medial prefrontal cortex and the posterior cingulate, and the left and right nucleus accumbens. All ROI data was extracted from the contrast of the reward stimulus versus the control stimulus. For further details, including the peak voxel coordinates chosen for the 6 mm spheres, see our previous work^18,20^.

### Statistical analysis

We used Bayesian statistics for all our analyses^50^. To determine the correlation between pupil dilation and symptom load (the main finding in the original study), we used a Bayesian modelling approach estimating the correlation^51^ using JAGS 4.3.1 in Matlab. JAGS is a software package that allows to generate and sample from probability distributions through the Markov chain Monte Carlo (MCMC) estimation algorithm^52^. Here, as in the original study^20^, we modelled the correlation and its posterior distribution between pupil dilation and number of depressive symptoms. We used the same model specifications as in the original study by sampling from a multivariate Gaussian distribution using uniform priors for pupil dilation (-2, 2), pupil dilation’s SD (0, 2) depressive symptoms (0, 20), depressive symptoms SD (0, 10) and the Pearson correlation coefficient (-1, 1). Furthermore, the following measurement uncertainty estimates were incorporated into the model. We computed the split-half reliability (trial 1–5 versus 6–10, resulting in a correlation of 0.87, see Figure S4) and used the published test–retest reliability value from the M-CIDI-interview (0.78)^47^ modelled as SD x sqrt(1-R_TEST-RETEST_).

For our replication analysis, we performed two additional control analyses. First, we modelled the correlation using a more conservative estimate for the standard measurement error estimates for the pupil data (0.70). Second, we used the differential mean pupil dilation score of the two stimuli associated with a behavioral response, i.e., reward and neutral stimulus (verbal feedback) to determine the correlation controlled for motor responses. In addition to the replication analysis, a combined posterior distribution was computed using both independent samples from the original and replication study together. Here, we analyzed the same correlation with uncertainty for the full sample, that is, depressed participants together with healthy controls of both samples. For all aforementioned analyses, the hypotheses were directional (the expectation was that the correlations were all negative as in the original work), which is indicated by the negative sign for the Bayes Factors (BF_(-)_). These BFs_(-)_ were obtained by dividing the number of samples below zero by the number of samples above zero of the posterior distribution.

For all the following extended analyses, we used the software package JASP 0.16.4.0 (https://jasp-stats.org/), which allows to perform Bayesian inferential statistics. We computed correlations between pupil dilation during reward anticipation and all 21 BDI-II items including the anhedonia score. To further study the specificity of our results, we computed the separate correlations between pupil dilation of the three stimuli reward, neutral and control (i.e., not the differential pupil score reward minus control) and number of depressive symptoms and anhedonia score. We then computed the correlations of pupil dilation with depressive symptom load for the other phases of the task, that is, fixation and reward consumption. For fMRI analyses, each of the seven ROIs was correlated with pupil dilation during reward anticipation, number of depressive symptoms, and the anhedonia score. In addition, we performed group comparisons of fMRI BOLD activity between acutely depressed participants and HC for all ROIs.

Bayesian analysis offers different possibilities to indicate and thus interpret the results, that is, either in favor of the null hypothesis (in our case, there is no correlation) or in favor of the alternative hypothesis. In this study, we report results for the above-mentioned analyses in relation to the alternative hypothesis indicated by BF_10_ (and BF_(-)_). In general, higher BF_10_ (and BF_(-)_) values mean stronger evidence provided by the data for the alternative hypothesis^53^. A BF_10_ around 1 indicates that the data is explained equally well by both null and alternative hypotheses. A BF_10_ between 1 and 3 provides anecdotal evidence, between 3 and 10 moderate evidence and between 10 and 30 strong evidence for the alternative hypothesis. In case BF_10_ exceeds 30, the data provides very strong evidence and BF_10_ above 100 indicates extreme evidence for the alternative hypothesis^50^.

### Supplementary Information

Supplementary Information.

## Data Availability

The data of this study are available from the corresponding author upon reasonable request.
