# Supplementary material for: Assessing hypo-arousal during reward anticipation with pupillometry in patients with major depressive disorder: replication and correlations with anhedonia
Source: Sci Rep. 2024 Jan 3;14:344. doi: 10.1038/s41598-023-48792-0 (PMC10764729; doi:10.1038/s41598-023-48792-0)
Supplement: Supplementary file 1 — Supplementary Information. [file 41598_2023_48792_MOESM1_ESM.pdf]

Supplemental Material

Assessing hypo-arousal during reward anticipation with pupillometry in patients with major depressive disorder: replication and correlations with anhedonia

Andy Brendler[1], Max Schneider[1], Immanuel G. Elbau[1,4], Rui Sun[1,5], Taechawidd Nantawisarakul[1], Dorothee Pöhlchen[1], Tanja Brückl[1], BeCOME Working Group[2], Michael Czisch[2], Philipp G. Sämann[2], Michael D. Lee[3], Victor I. Spoormaker[1]

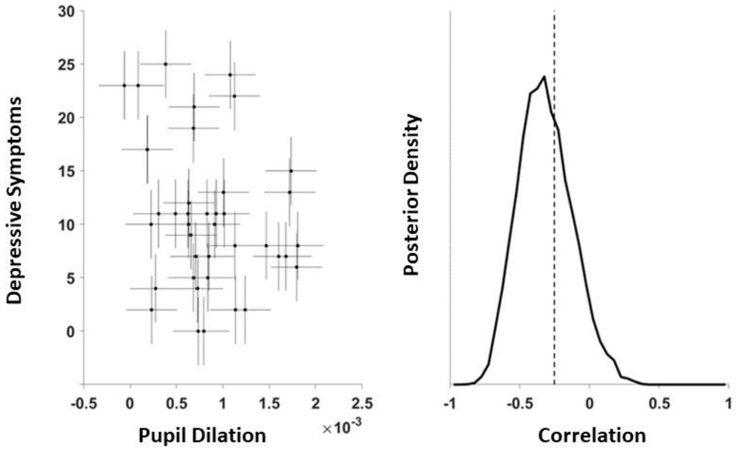

Figure S1. Correlation between pupil dilation (reward minus control stimulus) and the number of depressive symptoms with a more conservative uncertainty estimate in the replication sample. Instead of using the actual split-half reliability of 0.87, we also ran the analyses with a more conservative measurement uncertainty estimate for pupil dilation (test-retest correlation of 0.70). The correlation was $r = -0.31$, corresponding to strong evidence for $r < 0$ with a $BF_{(-)} = 15.4$. Each measurement including its measurement uncertainty (represented by vertical and horizontal error bars) is shown (left panel). The Bayesian model estimated the true correlation while accounting for measurement uncertainty by sampling from a multivariate Gaussian distribution, leading to a posterior distribution that indicates the likelihood of the modelled correlation (right panel).

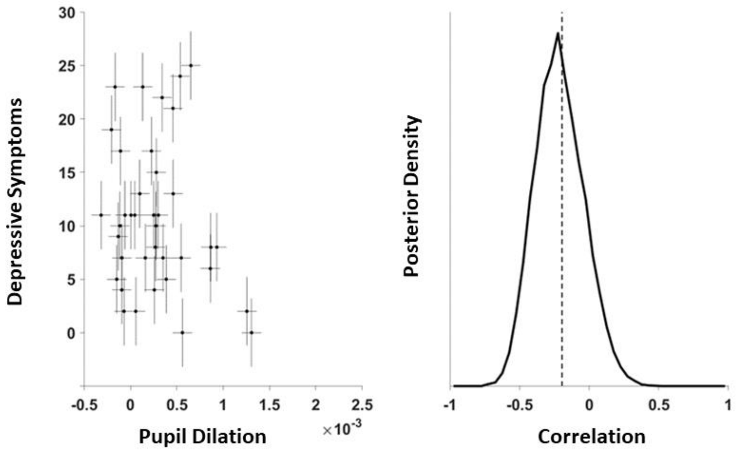

Figure S2. Correlation between pupil dilation (reward minus *neutral* stimulus) and the number of depressive symptoms. The correlation between the differential mean pupil dilation to the reward stimulus minus the pupil dilation during the neutral (verbal) stimulus and the number of depressive symptoms. The correlation was r = -0.21, corresponding to moderate evidence for r < 0 with $BF_{(-)}$ = 7.4, showing that the correlation still exists when comparing pupil dilation to the reward stimulus with another stimulus requiring a motor response and providing feedback about performance. Each measurement including its measurement uncertainty (represented by vertical and horizontal error bars) is shown (left panel). The Bayesian model estimated the true correlation while accounting for measurement uncertainty by sampling from a multivariate Gaussian distribution, leading to a posterior distribution that indicates the likelihood of the modelled correlation (right panel).

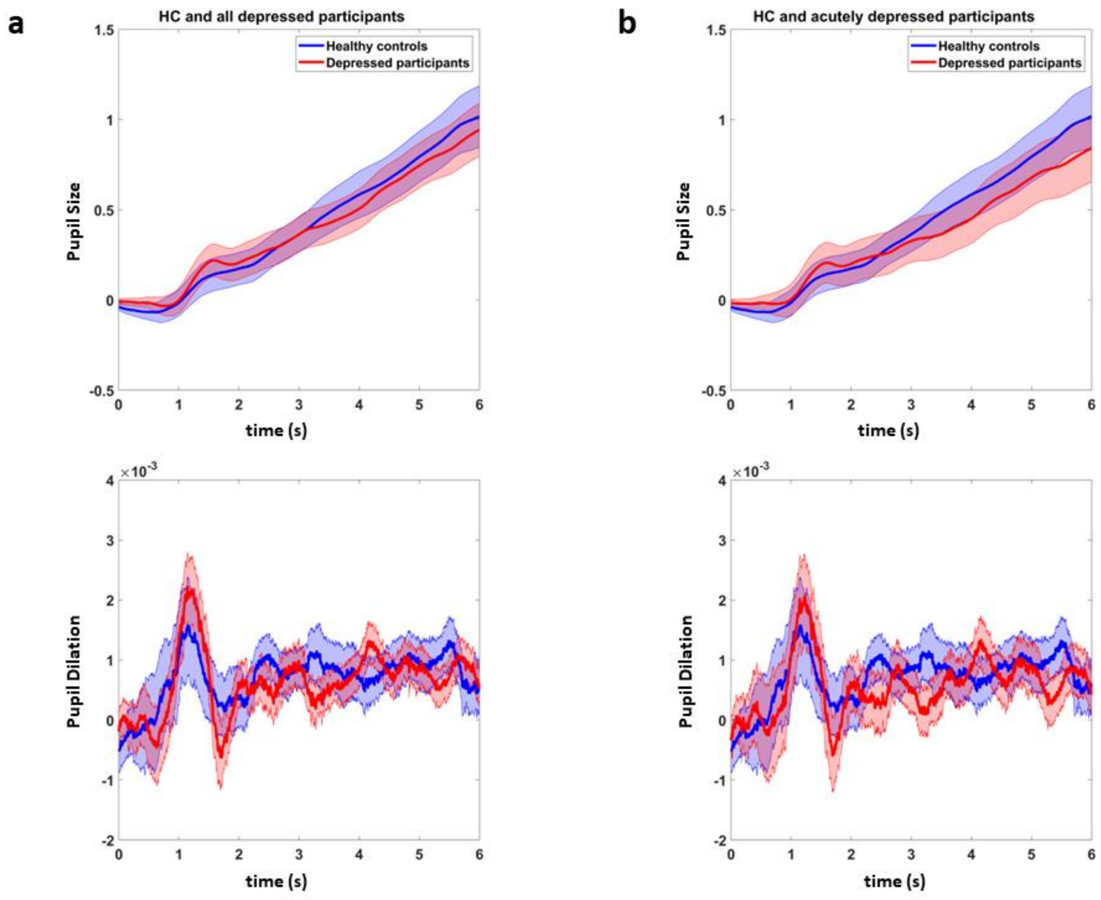

Figure S3. Group comparison of the time courses of pupil size and dilation over the 6s anticipation window for the reward stimulus. Shown for pupil size (**a**, top panel) and pupil dilation (**a**, bottom panel) for comparison between healthy controls and all depressed participants and for pupil size (**b**, top panel) and pupil dilation (**b**, bottom panel) for comparison between healthy controls and acutely depressed participants (five or more symptoms in the last two weeks). Mean time courses of all reward trials are shown including 95 % confidence intervals.

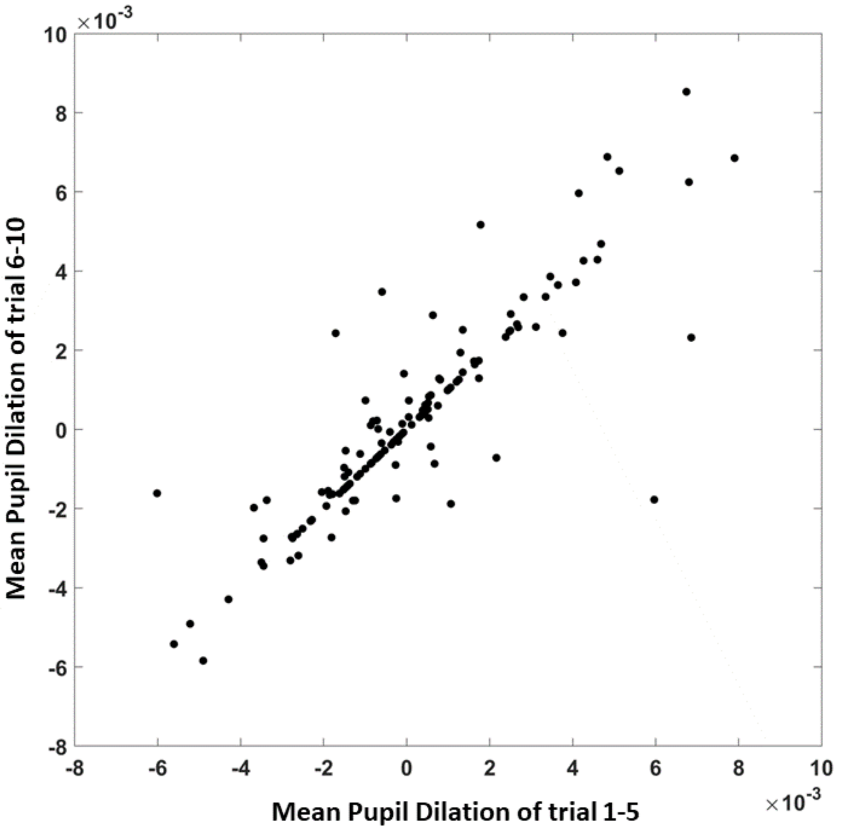

Figure S4. Split-half reliability of differential mean pupil dilation (reward minus control stimulus). The split-half reliability was calculated as the correlation between the differential mean pupil dilation values of trial 1-5 and trial 6-10 for all participants (0.87).
